# Is structural equation modeling applicable in a population health determinants assessment? An experience from the European Union

**DOI:** 10.1371/journal.pone.0337042

**Published:** 2025-12-11

**Authors:** Maciej Jankowiak, Justyna Rój

**Affiliations:** 1 Department of Organization and Management in Health Care, Poznan University of Medical Sciences, Poznań, Poland; 2 Department of Operational Research and Mathematical Economics, The Poznań University of Economics and Business, Poland; Military University of Technology Faculty of Civil Engineering and Geodesy: Wojskowa Akademia Techniczna im Jaroslawa Dabrowskiego Wydzial Inzynierii Ladowej i Geodezji, POLAND

## Abstract

The purpose of this study is to examine the relations between health determinants and overall health within population of the European Union by using the structural equation model, particularly given that not all health-determining factors have been theoretically identified. The significance of this issue stems, among other things, from the fact that the factors determining health are essential for policymakers for implementing an effective and accurate intervention in society, particularly considering that not all such factors have yet been theoretically identified. We hypothesized that (1) There is a statistically significant positive relationship between socioeconomic status and the health of the European Union population, (2) The structural equation model (SEM) is applicable for examining the complex interrelationships between socioeconomic status and health outcomes in the European Union population at the regional level. We used a dataset from EUROSTAT covering finally 258 regions at NUTS 2 in European regions for the year 2022 (or the nearest). We employed the SEM in this study due to its ability to simultaneously analyze multiple variables and latent constructs, thereby minimizing measurement error and enhancing the validity of the findings. So, our research has shown that a better social status of communities in European regions is associated with a higher level of health. Furthermore, a better economic situation of a region significantly improves the health of its inhabitants. However, in general economic factors have a stronger impact on health than social status. Thus, these findings have both theoretical and practical significance, as they identified key modifiable socioeconomic determinants of health and provided valuable insights for shaping effective public health policies and targeted interventions aimed at reducing health inequalities across European regions. Moreover, this study demonstrated the utility of SEM as a robust approach for examining complex relationships among health determinants including direct and indirect effects. By applying SEM, the research aligns methodologically with the growing body of literature in health sciences and contributes to a broader understanding of how socioeconomic factors influence health under varying regional conditions.

## Introduction

In recent years, “health” has become a subject of interest of researchers from other various disciplines than just medics and psychologists, due to an alarming increase in population health challenges, which suggests that some risk factors are not yet recognized or addressed by preventive strategies [1]. This is because no consensus has yet been reached on definitions of health and disease [2], while it is known fact, that the underlying processes leading to health are complex and therefore require investigation of interactions between several risk factors [1]. This is a significant problem because health is the most precious asset and widely regarded as the most valuable asset – a ‘resource for living’ allowing people to function and participate in many activities in society to pursue diverse life plans [3,4]. It implies that health holds dual significance: it is valuable on its own, and it also plays a crucial role in social progress and economic development. Therefore, health goals command a significant position on the United Nations 2030 Agenda for Sustainable Development [5].

The most cited definition of health is the one proposed by the World Health Organization (1948) that defines health as “a state of complete physical, mental and social well-being and not merely the absence of disease or infirmity” [6]. Despite remaining unchanged since 1948, the WHO’s definition of health has faced significant and ongoing criticism [7] and this is mainly due to its utopian character, conceptual pluralism as well as an unmeasurable nature [8–10]. Although many researchers have attempted to propose more modern and coherent definitions of health, a clear alternative to the WHO’s definition and a consensus on health definitions has yet to be established [2].

This complexity of health is also reflected by the production function of health, which was first described by Auster, Leveson and Sarachek (1969), who examined health (measured by mortality rate) as a function of both medical care and non-medical inputs [11]. Also, Lalonde (1974) identified four key components of health and emphasized the need for a holistic understanding of health highlighting the significance of socioeconomic factors [12]. Subsequently, numerous researchers have incorporated these approaches into their studies, while also introducing various other variables to explain health status, which are referred to in the literature as the socioeconomic determinants of health [13,14].

So, the World Health Organization proposes three main groups of health determinants such as: individual characteristics and behaviors, social and economic environment and physical environment. These determinants encompass key factors such as genetics, personal behaviors and coping skills, gender, income and social status, education, physical environment, social support networks and health services [15]. By shaping the conditions of daily life in which people are born, grow up, live, work, and age, these socioeconomic determinants affect people’s chances of achieving and maintaining good health. Therefore, many studies have focused on analyzing the relationship between a wide range of health outcomes and various socioeconomic elements, as well as identifying these determinants as the main root cause of many health inequities what was early discussed by Rój & Jankowiak in 2021 [16]. Determining the importance and scope of the impact of individual determinants is crucial for achieving health justice, and especially for developing actions and programs aimed at eliminating health inequalities [17]. Recognizing health inequities as a social justice issue and an ethical imperative, the World Health Organization (WHO) established the Commission on Social Determinants of Health in March 2005. This Commission presented its final report to WHO in July 2008 before concluding its work in which it urged governments and society to the social determinants of health and in creating better social conditions for health, particularly among the most vulnerable people [18]. Also, in 2010, WHO’s Commission on the Social Determinants of Health developed a widely used conceptual framework in purpose to explain the complex relationships between social determinants and health outcomes [19,20].

The good think is that these socioeconomic determinants are modifiable and can be influenced by social, political or economic processes as well as culture and norms, law, investment [21]. However, in the literature, it is also underlined that bringing about a reduction in their distribution inequities requires effective interventions in all sectors and therefore it is an significant challenge for health policies [22–24].

Thus, socioeconomic determinants of health have consequences for the economy, national security, business, and future generations [19]. As the Covid-19 pandemic has disproportionately impacted and exacerbated the current social determinants of health situation at individual and national, regional and global levels [25], therefore this study focus on these determinants in the context of their impact on the health outcomes.

However, the problem with the determinants of health is that some of them are observable such as blood pressure level, cholesterol level but there are some that are not directly observed (that are difficult to measure directly/ interconnected variables) such as lifestyle, socio-demography, and mental health condition or latent, which complicates the process of determining health outcomes [26]. Therefore, there is growing interest in the application of structure equation models (SEM), whose use has recently increased exponentially in health and medical sciences [27,28] as this model allows to handle latent variables (e.g., patient satisfaction, quality of life), test theoretical models, and analyze direct, indirect, and mediating effects [29].

So, the SEM is a set of statistical techniques used to measure and analyze the relations between variables, which can be latent and observed, or only between observed variables and their on each other [27,30,31]. It includes, among other techniques, the Linear Regression Model (LRM), Factor Analysis (FA), Confirmatory Factor Analysis (CFA), and Path Analysis [27].

The essence of the SEM is that it examines linear causal relationships among multiple variables simultaneously, while latent factors reduce measurement error. Thus, it is possible to “determine to what degree unknown factors influence shared error among variables - which may affect the estimated parameters of the model” [32].

This simultaneously ability to account for measurement error is its greatest advantage as the ability to manage measurement error is one of the greatest limitations of most methods [30,31]. Thus, it allows to test complex relationships simultaneously in one model rather than using multiple models [33]. Also, it makes SEM superior over other correlational methods such as conventional multiple regression analyses and in effect it has greater statistical power, which means lower probability of rejecting a false null hypothesis. Moreover, the SEM effectively manages missing data by using raw data rather than summary statistics. Therefore, it can be so useful method for a number of research designs including those analyzing the complex nature of disease and health behaviors, as it allows examination of both direct and indirect, as well as unidirectional and bidirectional relationships between measured and latent variables [32]. In fact, there are some studies, which have demonstrated that a structural equation modeling approach is particularly suitable for modeling the interrelationship between observable and unobservable factors that describe health status, such as: Boniface and Tefft [34], Chern et al. [35], Stafford et al. [36].

Even, Structural Equation Modeling (SEM) originated in the early 1900s, stemming from Spearman’s (1904) factor analysis and Wright’s (1918, 1921) invention of path analysis, however, the first introductory textbook on SEM wasn’t published until 1984. As computer programming advanced, researchers increasingly adopted SEM techniques in their studies. Today, SEM is recognized as “the preeminent multivariate technique” that is primarily and widely used in the social sciences in which it has its roots but then also increasingly used in epidemiology, public health, and the medical sciences [32–34].

Therefore, this study has adopted the structural equation modeling as a conventional technique that fulfills the requirements of the study. There are some of such studies, which focus on the impact of different range of socioeconomics determinants on health using SEM. However, these studies differ in the scope of analyzed determinants, health measures and spatial scope. So, Newton et al. (2024) used SEM to hypothesize a model of relationships between health determinants and outcomes within a region in the North of England using large-scale population survey data [37]. Also, Wang et al. (2019) analyzed the housing affects on health in China by using SEM [38]. Wirayuda (2020), used SEM in purpose to find how health status and resources (HSR), sociodemographic (SD) macroeconomic (ME) factors affect LE in Bahrain [39]. Another research of Wirayuda et al. (2022) also tried to understand how the sociodemographic (SD), macroeconomic (ME), and health-status and resources (HSR) factors affecting LE of population in Oman [40]. Then, Truong and Asare (2021) examined the effect of socio-economic features of low-income communities and COVID-19 related cases in New York City [41]. There are some specifically focusing on the various dimensions of HRQOL and its relationship with various sociodemographic characteristics, functional status and disease activity using a structural equation modeling (SEM) approach in patients with Rheumatoid Arthritis in Southern India [42]. Mosallanezhad et al., (2017) designed their research to evaluate the link between socioeconomic status, physical activity, independence and the health status of older people in Iran by using structural equation modeling [43].

Therefore, the aim of this study is to examine the relations between health determinants and outcomes within European population by using the structural equation model. The hypotheses are as follows:

1. There is a statistically significant positive relationship between socioeconomic status and the health status of the European Union population.
2. The structural equation model (SEM) is applicable for examining the complex interrelationships between socioeconomic status and health outcomes in the European Union population at the regional level.

In order to verify these hypotheses, we used the database of EUROSTAT [44], which determined the final range of socioeconomic variables adopted for the study and the year of research, which is 2022 (or nearest). Thus, it was possible to derive the data at the NUTS 2 level, which ensures the analysis of basic regions of European Union member states and Switzerland. Hence, our research fills an existing gap by providing more specific information on the spatial diversity of the European population in terms of the socioeconomic determinants of health. The novelty of this research also arises from it being the first time the structural equation model application in a such range of data and spatial scope allowing for the design of regional politics. Thus this study contributes to the research area of socioeconomics health determinants and outcomes and would improve the understanding of the factors, which are associated with health of European Union population. Also, the obtained results may be beneficial for such parties as government, policymakers, as they can support the health and social policies.

This study is structured as follows that this introduction with theoretical arguments for this research constitutes the Introduction section; the Materials and Methods section described the data and methods used in this article; then the results are shown in the Results section and theoretical and practical implications are presented in the Discussion section; the lessons from this article were drawn in the Conclusions section.

## Materials and methods

### Scope of the study and dataset

In the study the influence of socioeconomic status of European regional populations on their health outcomes was quantitatively examined. Because both socioeconomic status and health are difficult to evaluate using a single indicator, the structural equation modeling (SEM) was implemented. In SEM methodology used in the study socioeconomic status and health outcomes are treated as latent variables not seen directly but measured using aggregates of several commonly available statistical indicators.

Data for the year 2022 (or 2021 if 2022 was unavailable) was derived from the EUROSTAT database. Data was collected at the level of NUTS 2 (the Nomenclature of Territorial Units for Statistics) covering basic regions of 27 member states of the European Union, Switzerland and Norway (countries of European Free Trade Association), and Serbia (the European Union candidate country). The geographic scope of the study was determined by accessibility of published data. Regions for which obtaining information on at least one indicator for one latent variable failed were excluded from statistical analysis. Finally, 258 basic regions were involved into the SEM procedure. The underlying data from all of these 258 European regions used for the further variable descriptive statistics calculation and the analysis of SEM were presented in S1 Appendix.

The study was conducted using aggregated data originating from official statistical publications. There was no inclusion of individual human participants into the study, therefore an ethical approval and a privacy protection were not required.

### Measurable variables

Five manifest variables were used in order to assess a socioeconomic status of inhabitants of European regions. These variables were: level of education, Internet usage, unemployment, risk of poverty and regional gross domestic product.

Level of education (EDU) was measured using the EUROSTAT tertiary educational attainment indicator. This indicator showing the percentage of the population in age of 25–64 years old who completed high studies is based on the EU Labour Force Survey.

An assessment for Internet usage (INT) was the indicator of individuals regularly using the Internet which shows the percentage of persons who use the Internet at least one a week.

Unemployment (UNEMP) was assessed using the EUROSTAT unemployment rate indicator. This indicator shows unemployed persons as a percentage of economically active population.

Risk of poverty (POVERT) was evaluated using the EUROSTAT at-risk-poverty rate by NUTS 2 region. This indicator shows the persons with an equivalised disposable income below 60% of the national median equivalised disposable income as a percentage of the total population.

Regional gross domestic product (REG-GDP) was calculated using the EUROSTAT regional gross domestic product by NUTS 2 region indicator. In our dataset regional GDP was expressed in thousands PPS (purchasing power standards) per one inhabitant of a NUTS 2 region. Usage of PPS (concept similar to purchasing power parity) instead of EURO eliminates differences in prices between compared countries.

Health status of European regions residents was judged using three manifest variables: life expectancy and mortality rates due to malignant neoplasms and ischaemic heart disease. Life expectancy at birth (LIFE-EXP) was originated from the EUROSTAT database by NUTS 2 regions and indicates the mean number of years that a newborn child (both female and male) can expect to life.

Mortality rate due to neoplasms (CANCER) refers to the EUROSTAT death due to cancer by NUTS 2 region indicator and shows deaths caused by all malignant neoplasms per 100,000 inhabitants.

Mortality rate due to ischaemic heart disease (IH-DIS) is based on the EUROSTAT death due to ischaemic heart diseases by NUTS 2 region indicator which presents all deaths caused by reduced blood supply to the heart (mainly by myocardial infarction) per 100,000 inhabitants.

Descriptive statistics of above mentioned measurable variables is presented in Table 1. Normality of variables distribution was assessed using the Kolmogorov-Smirnov test. Only three variables (EDU, INT and CANCER) have got a normal distribution (Kolmogorov-Smirnov p-value above 0.05).

**Table 1:** Descriptive statistics of measurable variables.

Variable	Valid N	Mean	Median	Variance	Std.Dev.	Skewness	Kurtosis	Kol-Smi
EDU	258	33.64	33.00	109.94	10.49	0.34	−0.49	p > 0.05
INT	176	88.97	89.69	34.75	5.90	−0.70	0.30	p > 0.05
UNEMP	252	6.31	4.85	18.39	4.29	1.90	5.11	p < 0.05
POVERT	238	16.48	14.80	46.21	6.80	1.13	1.19	p < 0.05
REG-GDP	251	33.61	31.10	174.52	13.21	1.55	4.41	p < 0.05
LIFE-EXP	258	80.50	81.30	7.06	2.66	−0.95	0.09	p < 0.05
CANCER	252	241.61	237.29	941.59	30.69	0.76	0.38	p > 0.05
IH-DIS	252	125.13	97.21	8704.06	93.30	1.70	2.54	p < 0.05

Valid N – number of valid observations; SD – standard deviation; Kol-Smi – p value in Kolmogorov-Smirnov test.

### Structural model

The structural model consist of three latent variables. Two of them are exogenous. These are the social status and the economic status. The third latent variable, the health status is endogenous, explained by previous mentioned two exogenous variables.

**Fig 1:**
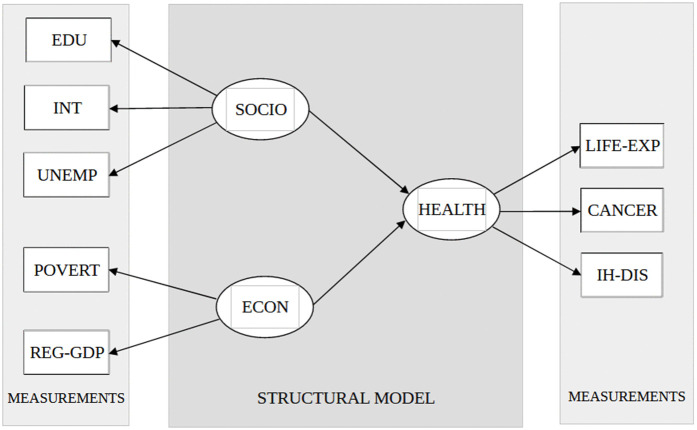
Path diagram of the structural equation model. Abbreviations explained in the text.

The social status (SOCIO) is measured by three manifest variables: EDU, INT and UNEMPL. The economic status (ECON) is measured by two manifest variables: REG-GDP and POVERT. The endogenous latent variable, the health status (HEALTH) is measured by three manifest variables: LIFE-EXP, CANCER and IH-DIS. The structural model is substantive in its nature, based on theoretical consideration presented in the Introduction section. The path diagram reflecting the structural model and its measurements is shown at Fig 1.

### Statistics

Estimations of the model parameters were done using the asymptomatically distribution free estimator (ADF). This estimator was chosen due to its resistance to deviation from multivariate normality that is important according to non-normal distribution of majority of the observed variables. The model goodness of fit was evaluated using three parameters: Chi square statistic, the root-mean-square-error of approximation index (RMSEA) and the goodness-of-fit index (GFI). Calculations were done using STATISTICA software (TIBCO Software Inc. 2017, Statistica data analysis software system, version 13).

## Results

Results of estimation of the model coefficients are presented in Table 2. In the structural model the direction of effects of the exogenous socioeconomic status on the health status (treated as endogenous) is positive, which was expected. The coefficient for the SOCIO → HEALTH path is 0.53 (p < 0.001) that indicates significant positive impact of the good social status of a local population on its level of health. Similarly the coefficient for the ECON → HEALTH path is positive and significant as well (0.85, p < 0.001) which confirms relevant advantageous influence of better economic situation on population health.

**Table 2:** Results of the model calculations.

	Coefficient	Standard error	p-value
**Measurement model**			
**Social status (SOCIO)**			
- EDU	6.51	0.66	< 0.001
- INT	3.65	0.35	< 0.001
- UNEMP	1.34	0.20	< 0.001
**Economic status (ECON)**			
- POVERT	−2.01	0.32	< 0.001
- REG-GDP	3.86	0.61	< 0.001
**Health status (HEALTH)**			
- LIFE-EXP	2.75	0.11	< 0.001
- CANCER	−19.59	1.98	< 0.001
- IH-DIS	−102.71	6.41	< 0.001
**Structural model**			
**Effects on health status (HEALTH)**			
- SOCIO	0.53	0.06	< 0.001
- ECON	0.85	0.03	< 0.001

In the measurement model coefficients indicate relations between measurable and latent variables. All these coefficients are significant (p < 0.001). The relation of the economic status to the regional GDP is positive (coefficient 3.86) and otherwise to the risk of poverty is negative (coefficient −2.01). Relations between the social status and all of its three measurable variables: education level, internet access and, surprisingly, unemployment rate are positive (coefficients are respectively 6.51, 3.65 and 1.34).

Relations between the health status and its measurements depend on nature of the measurable variable. In case of the life expectancy the correlation is positive (coefficient 2.75). Mortalities due to neoplasms and ischaemic heart disease are correlated negatively (coefficients respectively −19.59 and −102.71).

Goodness of a fit of this model is not excellent. Chi square statistics equals 121.5 and its ratio to df = 18 is 6.75, much more than 3.00 and less indicating a good fit. RMSEA index is 0.19 (90% CI 0.16–0.23) when a good fit needs value below 0.10. GFI index is 0.85, which is below 0.90 needed for a good fitting. Unfortunately, unlike in experimental researches, our study is based on available published macroeconomic data and possibilities of the model optimization are limited by accessible information. The issue of improvement of the model fit goodness is wider talked over in the Discussion section.

## Discussion

In this section, we discuss the implications of our findings, compare them with previous research, and suggest potential directions for future studies and practical applications. First and foremost, the results obtained through structural equation modeling (SEM) confirm the hypothesis of a positive association between socioeconomic status and population health. Specifically, this analysis revealed that both social status and economic status exert a statistically significant and positive influence on health. In such, the hypothesis about applicability of the structural equation model (SEM) has been positively verified as the complex interrelationships between socioeconomic status and health at the regional level in the European Union population has been recognized.

These findings have emphasized the utility of structural equation modeling as a powerful tool for estimating hypothetical relationships among latent constructs across multiple levels within a developed model, which is also aligned with some other research [30,45–47]. They showed also that SEM is useful tool in identifying the direction and significance of relationships between constructs such as socioeconomic status and health disparities [48–50]. This way, by applying structural equation modeling (SEM), this study aligns methodologically with the growing body of research in this field. However, the specific combination of the type and scope of variables, as well as the spatial coverage in individual studies [37–39,42,43], makes direct comparison of results difficult. Nevertheless, it contributes to a broader understanding of how socioeconomic factors influence health under different conditions – specifically, in this case, within the population of European regions. The most important, that this study confirmed the existence and direction of the relationship between socioeconomic variables and health outcomes.

Socio and economic status are a complex concept that may influence health both directly or indirectly through related. They encompass such functions as wealth, income, educations, while also reflecting one’s position or rank within a specific social hierarchy. So, our research has shown that a better social status of communities in European regions is associated with a higher level of health. Furthermore, a better economic situation of a region significantly improves the health of its inhabitants. However, in generally economic factors has a stronger impact on health than social status.

In detailed, this research showed that first, a higher level of education contributes to better health. The results of our study are consistent with the widely recognized theoretical and empirical relationship between education and health. This positive link between education and health, originally conceptualized in Grossman’s health demand model is a crucial connections in health economics that’s because education enhances individuals’ cognitive abilities, decision-making skills, and self-efficacy, while also fostering a sense of control that encourages healthier behaviors and lifestyle choices [51–54]. Then numerous empirical studies have indicated that educational attainments are linked to better health [55–57]. Then, greater access to the internet is also linked to better health, which may result from improved access to health information and increased availability of medical services as telemedicine enables remote consultations, which is especially important in rural or underdeveloped regions, which is also widely documented by empirical research [58]. However it is interesting that unemployment also shows a positive impact on health, which may be surprising as opposite relations are rather presented by the theory [59,60]. However such positive relation is possible as unemployment can be correlated with other social factors such as social support or demographic structure. Also, in the literature it is pointed out that unemployed people may be more likely than employed people to visit physicians, take medications, or be admitted to general hospitals, which can positively influence their health [61]. Also, unemployment provides a temporary break from a stressful job, allowing for the restoration of mental balance, reflection on one’s professional life, and more time for physical activity and social relationships. Sometimes employment can lead to occupational exposure to factors harmful to health, and to development of occupational diseases, which worsen the population health status. In addition, unemployment benefits may offset the negative health consequences of joblessness [62,63].

As for economic factors, our findings showed that higher poverty levels worsen health, while a higher level of economic development supports better health. This is consistent with both theoretical frameworks and prior empirical findings as increases in absolute income levels often signal macroeconomic growth, which can expand access to public services such as education, healthcare, and social security – ultimately improving population health as well as it can also result in environmental health poverty [64].

Regarding the health status construct, it is positively associated with life expectancy and negatively associated with mortality due to cancer and ischemic heart disease, reflecting the multidimensional nature of health. So, the longer the life expectancy, the better the overall health status, while higher cancer mortality worsens the health assessment as well as there is a strong negative impact of heart disease on overall health. Therefore, this implies addressing major health challenges such as cancer and cardiovascular diseases.

These findings by identifying these variables highlights their role as risk factors in the emergence of health inequities. Also, this research demonstrates the value of structural equation modeling for the examination of health determinants and outcomes.

The results obtained have both theoretical and practical significance. By identifying the significance and direction of the health determinants, these findings contribute to research on factors influencing health, which are of particular importance in light of evidence from the literature [1] indicating that not all such factors have yet been fully identified and then which is especially important given the value of health for both individuals and the economy. Also, this study contributes to the growing body of research employing structural equation modeling (SEM), reflecting the increasing interest in its application within health and medical sciences, which stems from the fact that SEM enables the examination of direct, indirect, and mediating effects, and is therefore particularly well-suited to capturing the complex and multidimensional nature of health-related factors.

The practical implications of these findings stem from the fact that they address the socioeconomic determinants of health, which are modifiable. At the same time, disparities in these determinants may lead to inequalities in health outcomes. Therefore, accurate identification and assessment of these determinants is essential for the development effective recommendations for health policy particularly these aimed at populations in EU countries. The detailed relationships identified in this study along with the proposed model, have the potential to influence government’s decision to address predisposing factors contributing to health inequalities. This study provides valuable insights for designing targeted health interventions. The nature of these determinants and their significance for health imply that, in practice, it is essential to ensure that public policy and health policy mutually inform and reinforce each other.

Therefore, these findings could have direct implications for public health practice and policy, as they offer a clear evidence-based foundation for designing interventions that address health inequalities. Based on the identified relationships between various determinants and population health, a number of public policy recommendations can be proposed. For example, health education should be integrated into school curricula from an early age, and adult learning programs should be expanded to provide free courses and training in areas such as healthy lifestyles, nutrition, stress management, and disease prevention. It would be also critical to improve digital infrastructure in rural and marginalized areas. Expanding access to the internet enables the development of telemedicine services and ensures broader access to reliable online health information. Such information should also be adapted to the needs of older adults and those with lower levels of education, for example through simplified content and accessible formats. Then, regional development policies should prioritize investment in infrastructure, education, and healthcare services in less developed areas to reduce disparities. It would be also worth to integrate health policy with social policy along with the establishment of cross-sectoral public health teams that bring together experts in health, education, technology, economics, and social policy. In addition, national programs for the prevention of cardiovascular diseases and cancer should be strengthened or further developed. These programs should emphasize early diagnosis, healthy lifestyle promotion, and control of risk factors. Public funding should support screening initiatives and educational campaigns addressing diet, smoking, physical inactivity, and other modifiable risks. Moreover, improving the quality and availability of regional data is essential for tailoring policies to specific local needs and enhancing their effectiveness.

This study is not without limitations. The most important one, stems from the fact that the empirical research is based on publicly available data rather than experimental research. As a result, the potential for model optimization is inherently constrained by the scope and level of detail of the available data. Also, some limitations imply from the weaknesses of the SEM as like any method, it has also its limitations. While latent variables are a closer approximation of a construct than is a measured variable they may still fall short of being entirely pure indicators. Also, their variance may include not only the true variance of the observed concept but also shared measurement error among them. Although, the advantage of SEM lies in its ability to analyze multiple variables simultaneously, this benefit often requires larger sample sizes to maintain the reliability and validity of the results.

Despite these promising results, the model’s overall fit indices suggest space for improvement. Goodness of fit of the model is not too perfect. Except the chi-square test which limitations are discussed in the literature [65], also other fit indices (RMSEA and GFI) showed at most mediocre goodness of fit. We faced challenges in improvement of the model fit. There were two main reasons of this fact. The first reason is missing data. We included to the research 258 European regions for which data deficiencies were tolerable according to the study assumptions, but the number of regions which had got a full dataset was substantially smaller. Only 156 of them had no missing values. Missing values can lead to decreasing a sample size (due to exclusion of these items which data deficiencies are too large), and finally to deterioration of a model estimations accuracy [66]. Our research was not experimental (which allows for designing appropriate sample size), but restricted to published statistical data. Releasing of more complete datasets by national and international statistical offices (like EUROSTAT) would enable a further improvement of our model.

The second reason is probably more essential. Among dozens of socioeconomic and health indices published by EUROSTAT almost all are at a national level. Only a few indices are at a regional level. National level, where study units consist of not regions, but entire countries, generates to small study sample to apply many intricate statistical methods including SEM. Restriction of a variable choice to only several indices leads to limitation of more complex model constructing. Additionally, too narrow choice of variables increases a risk of misspecification of the model due to lack of appropriate indices reflecting real factors behind theoretical considerations [67].

Due to its mediocre goodness of fit our model has got rather weak predictive abilities. Nevertheless, it can play a certain role in exploring substantive processes underlying theories of health determinants and estimation of „the operating model” [68]. Improvement of the model, based on both better quality of statistical data and a development of new analytical techniques used in structural modeling [69], can lead to more complete explanation of real health determination processes.

Besides, another limitation arises from the fact that the analysis was conducted using aggregated regional-level data and thus they cannot fully capture the complexity of SES-health interactions at the micro level. Micro-level analyses would allow for more precise policy recommendations and better identification of vulnerable subpopulations. Moreover, the use of regional averages may obscure important within-region variations as relations observed at the group level do not necessarily reflect individual-level relationships. For example, the unexpected positive association between unemployment and health may be confounded by contextual factors such as welfare policies, demographic structure, or access to healthcare services, which vary significantly across EU regions. These factors may distort the interpretation of results therefore there is a need for caution when drawing conclusions from aggregated data.

Regarding to the direction of future studies, they would greatly benefit from more comprehensive datasets, ideally collected and made available by institutions like EUROSTAT, which will allow to accomplish a perfect model with more data collected over time. Expanding the scope of such data would enable more accurate modeling and deeper insights into socioeconomic and health-related dynamics. Then, as such, the factors determining health are crucial for policymakers to study in order to implement effective and accurate interventions in society, therefore a promising direction for future research would be to conduct in-depth analyses at the level of individual countries, allowing for a more detailed understanding of how national contexts influence health outcomes. Also, in light of the findings on the impact of unemployment, it would be valuable to explore the extent to which factors such as social support and the amount and accessibility of unemployment benefits, demographic structure, and access to healthcare services mediate the relationship between unemployment and social status and health. Moreover, future research could also focus on refining the theoretical constructs used in the model by developing more precise indicators for complex concepts such as socioeconomic status or social support. Incorporating multidimensional measures and validating them across different contexts would enhance the robustness of future models but it could also reduce the risk of misspecification and improve the explanatory and predictive power of structural analyses.

## Conclusions

The study is the first attempt to compile all different aspects of health comprehensively in European regions with application of structural equation modeling (SEM). These findings showed that population with higher social status across European regions tend to experience better health. In addition, economic status also plays a significant role in enhancing the health of the population. However, economic conditions appear to have a stronger influence on health than social status. Thus, the findings highlight the significant role of socioeconomic factors – particularly education, economic development, and digital access – in shaping population health, while also revealing nuanced effects such as the potentially positive health impact of unemployment under certain conditions. In addition, by applying SEM, we demonstrated the model’s effectiveness in capturing complex interdependencies between social, economic, and health-related variables. Overall, this research contributes to a deeper understanding of how socioeconomic factors shape health disparities and supports the use of SEM in public health policy analysis. Although the model’s predictive power is limited due to data constraints and only moderate goodness of fit, it still offers valuable insights into the mechanisms behind health disparities. The study underlined the strong need for more comprehensive and regionally detailed datasets to improve model accuracy and policy relevance. Despite its limitations, the research contributes to the growing body of literature using SEM in health sciences and provides a foundation for future studies aimed at refining models and informing targeted public health interventions. Ultimately, understanding and addressing the socioeconomic roots of health inequalities remains essential for effective policymaking in the European context.

## Supporting information

S1 Appendix Summary of data used in calculations. (XLSX)
